# Characterizing the rod pathway in cone-dominant thirteen-lined ground squirrels

**DOI:** 10.3389/fopht.2023.1271882

**Published:** 2023-11-16

**Authors:** Riley Ferguson, Kiyoharu J. Miyagishima, Francisco M. Nadal-Nicolas, Wei Li

**Affiliations:** Retinal Neurophysiology Section, National Eye Institute, National Institutes of Health, Bethesda, MD, United States

**Keywords:** AII-amacrine, bipolar cells, thirteen-lined ground squirrels, scotopic vision, rod pathways

## Abstract

AII-amacrine cells (AIIs) are widely accepted as a critical element of scotopic pathways mediating night vision in the mammalian retina and have been well-characterized in rod-dominant mice, rabbits, and non-human primates. The rod pathway is characteristic of all mammalian eyes, however, the anatomic and physiologic role of AIIs and the rod pathways in cone dominant thirteen-lined ground squirrels (TLGS) is limited. Here, we employed both immunohistochemistry and electrophysiological approaches to investigate the morphology of AIIs and functional aspects of the rod pathway in TLGS. In all TLGS retinas examined, putative AIIs were calretinin-positive and exhibited connections to rod bipolar cells with decreased cell density and expanded arborization. Notably, AIIs retained connections with each other via gap junctions labeled with Connexin36. Comparisons between single photoreceptor recordings and full-field electroretinograms revealed scotopic ERG responses were mediated by both rods and cones. Thus, the components of the rod pathway are conserved in TLGS and rod signals traverse the retina in these cone-dominant animals. AIIs are sparsely populated, matching the diminished rod and rod bipolar cell populations compared to rod-dominant species. The infrequent distribution and lateral spacing of AII’s indicate that they probably do not play a significant role in cone signaling pathways that encode information at a finer spatial scale. This contrasts with the mouse retina, where they significantly contribute to cone signaling pathways. Therefore, the AII’s original function is likely that of a ‘rod’ amacrine cell, and its role in cone pathways in the mouse retina might be an adaptive feature stemming from its rod dominance.

## 1 Introduction

By surviving the ‘nocturnal bottleneck’, the mammalian retina evolved an extraordinary rod system enabling superb night vision and which piggybacks onto the cone pathway, suggesting an evolutionary adaption with additional neural circuitry elements (1, 2). AII amacrine cells (AIIs) have been identified as an essential component of the mammalian rod pathway to convey rod signals to cone pathways. In mice, rabbits, and non-human primates, these cells have been rigorously investigated, and found to deliver both ON and OFF rod signals to the corresponding cone bipolar cells via gap junctions and glycinergic inhibitory synapse, respectively. AIIs have also been implicated in regulating the transition from scotopic to photopic vision (3). In addition, they perform robust cross-inhibition between ON and OFF systems involving cone bipolar cells and ganglion cells, thus, it has been well established in rod-dominant species that AIIs, in addition to signaling rod signals, play an integral role in the cone signaling pathway. However, the morphology and function of AIIs in the retina of cone-dominant species remains unclear. Specifically, it is unclear whether AIIs are only evolved in rod-dominant species to serve rod signaling functions, or if they are an essential piece of the conserved cone pathways that predate the evolution of enhanced rod pathways.

The thirteen-lined ground squirrel (TLGS) is one of the rare cone-dominant mammalian species. It has been utilized to study retinal physiology and ocular pathologies for its unique retinal features (4, 5) It has proved to be a superb model for studying cone photoreceptor physiology, including cone synapse and cone pathways (6, 7) however, its rod system has not been extensively explored (8). The AIIs in the ground squirrel retina may provide a clue for its role in cone pathways in the context of evolution. Better understanding of their visual pathways and anatomy may yield insight into the translational value of these models. Notably, the universal presence of scotopic vision in another cone-dominant species, the California ground squirrel has been called into question when data revealed a subset of *Spermophilus beecheyi* without functional scotopic vision (9, 10). In *Ictidomys tridecemlineatus* or TLGS, functional scotopic vision has not yet been fully explored. Past studies have identified scotopic vision using relative spectral comparisons of electroretinography, but the cone threshold of light detection has yet to be established for TLGS. This paper aims to extend the limited literature on rod vision of the TLGS with new data on the anatomy of AIIs and the functional measurements of rod vision in this cone-dominant retina. Better understanding of the visual pathways and anatomy of TLGS may yield insight into the translational value of these models.

## 2 Methods

### 2.1 Animal handling, anesthesia, and analgesia

A colony of thirteen-lined ground squirrels (TLGS) were obtained from a breeding colony at University of Wisconsin, Oshkosh ~200g, n=6 animals (3 male, 3 female) and kept in a temperature and light controlled room with 12:12 hour light to dark cycle. Animals were allowed food and water access ad libitum. TLGS were treated and maintained according to their protocol (ASP#595) approved by the National Institutes of Health guidelines for Animal Care and Use Committee in research and by the Ethical and Animal Studies Committee of the National Eye Institute. All animals were compliant with the Statement for the Use of Animals in Ophthalmic and Vision research of the Association for Research in Vision and Ophthalmology (ARVO). We adhered to all laws and regulations set forth by the United States and the United States Department of Agriculture.

Animals undergoing electroretinography (ERG) were provided anesthesia using inhaled isoflurane (Fluriso, Vet One, United Kingdom). During procedures artificial tears (Systane Ultra, Norvartis, Alcon) were applied to maintain corneal surface hydration.

### 2.2 *In vivo* TLGS electroretinogram recordings

Six TLGS, three of each sex, were dark-adapted for eight hours in their home cages. Anesthesia was induced with Isoflurane at 5% and maintained at 3% during the procedure. The TLGS recorded were in their awake, nonhibernating state. 1% Tropicamide drops (Akorn Inc., Lake Forest, Illinois) were applied to both eyes to induce mydriasis. The ERGs were obtained with the Diagnosys (Diagnosys LLC, Lowell, Massachusetts) system using their ColorDome LabCradle stimulator and traditional gold wire electrodes placed over both eyes. The system’s integrated self-regulated heater warmed the animals continuously to 37°C to maintain consistent body temperature. All ERGs were recorded under red-light conditions in a darkroom.

A drop of 1% carboxymethylcellulose was placed on each eye and encased both the electrode and the corneal surface on both sides.

ERG responses were elicited by brief xenon white flashes (4 ms) presented at varying intensities (cd sec/m^2^): 0.1 (5 flashes), 0.3 (5 flashes), 1 (5 flashes), 3 (4 flashes), 10 (3 flashes), 30 (3 flashes), 100 (3 flashes), 300 (3 flashes), 600 (3 flashes), 1000 (3 flashes), 3000 (3 flashes). The parameters controlling the light stimulus were written in and controlled using the Espion V6 software (Diagnosys LLC, Lowell, Massachusetts). Data were then stored and analyzed using a custom program written in MATLAB R2017a (Mathworks, Natick, Massachusetts RRID : SCR_001622). Estimates of equivalent light intensity expressed as photons/µm^2^ were calculated using a custom program in MatLab from measured optical power values obtained with a handheld digital power meter console PM100D (ThorLabs, Newton, New Jersey). The emission spectrum of the xenon stimulus was provided by Diagnosys, obtained by running the ColorDome at 3 cd sec/m^2^ at 30Hz for several seconds while taking measurements with a UV-VIS spectrometer. The photoreceptor spectra for the TLGS were estimated from published spectral sensitivity curves (11).

### 2.3 ERG light conversion from cd·sec/m^2^ to photons/µm^2^

The wide spectrum, white xenon lamp was used as the photic stimulator for TLGS ERGs because both the blue and green LEDs were not sufficiently bright enough to saturate the photoresponse. The power was measured for each stimulus light intensity using a calibrated photometer (PMD100D, Thorlabs) with a detector size of 94,090,000 µm^2^ and were converted to equivalent 525 nm photons by convolving the power-scaled spectral output of the xenon flash (provided by Diagnosys LLC) with the normalized TLGS spectral sensitivity curve digitally extracted from Jacobs et al., 1985 using PlotDigitizer (https://plotdigitizer.com/app) (11). The TLGS spectral sensitivity curve in Jacobs et al., is corrected taking into consideration the relative absorbance curves for the lens and corneas (12). The TLGS spectral sensitivity curve from Jacobs et al., can also be further corrected by multiplying by the cumulative spectral transmittance at the retina in the TLGS (12). These estimates make it possible to calculate the stimulus light intensity in photons/µm^2^. The calibrated photon flux (photons per µm^2^ per second) values were then multiplied by the stimulus duration (0.004) to convert to photons/µm^2^.

### 2.4 Tissue preparation

The 6 TLGS used in the electroretinogram study were then euthanized humanely using inhaled carbon dioxide. The retinas were dissected from each eye and fixed in 4% paraformaldehyde in a 0.1 M phosphate buffer for 1 hour each and then stored in phosphate-buffered saline (PBS). Additional retinas were cryopreserved using a graded sucrose series (Sigma, #84097) and embedded in optimal cutting temperature (OCT) compound (Sakura Finetek, Torrance, CA, USA) at −80°C for cryo-sectioning (Leica, CM3050S) at 16 μm thickness.

### 2.5 Immunofluorescence

Each retina was washed with 0.1% Triton in PBS for ten minutes four times for tissue permeabilization. Each retina was then incubated with calretinin (AB1550, Millipore; 1:5000), Protein Kinase A, regulatory subunit IIB (AB_610626, BD Transduction Laboratories; 1:500), and PKC-alpha antibody (AB_397514, BD Transduction Laboratories; 1:500) overnight at RT. The retinas were again washed with 0.1% Triton in PBS for ten minutes four times and again stained overnight with secondary antibodies: donkey anti-rabbit Cy-3 (1:200), donkey anti-mouse Cy-5 (1:200; Jackson ImmunoResearch Laboratories), and donkey anti-goat Alexa-488 (1:200; Molecular Probes). Neurobiotin was visualized by Alexa-488– conjugated streptavidin (Molecular Probes). Each retina was again washed 4x with PBS and then mounted onto glass microscope slides using antifading mounting medium with their vitreal side up.

### 2.6 Single cell recordings of rod, M-, and S-cones

Experiments were conducted on approximately 1-year-old ground squirrels. Upon euthanization, one eye was removed from a ground squirrel, and the retina was carefully isolated under infrared illumination. The isolated retina was placed in Locke’s solution, consisting of 112.5 mM NaCl, 3.6 mM KCl, 2.4 mM MgCl2, 1.2 mM CaCl2, 3 mM Na2-succinate, 0.5 mM Na-glutamate, 0.02 mM EDTA, 10 mM glucose, 0.1% MEM vitamins (M6895; Sigma-Aldrich), 0.1% MEM amino acid supplement (M5550; Sigma-Aldrich), 10 mM HEPES, pH 7.4, and 20 mM NaHCO3. Next, the retina was divided into 6 pieces, with one piece used immediately for recording while the others were stored in Locke’s solution bubbled with 95% O2/5% CO2 at room temperature for up to 10 hours. Each piece was then carefully chopped into 20-25 small fragments on a Sylgard plate (24236-10; Electron Microscopy Sciences) using a razor blade, all performed within Locke’s solution. Subsequently, the tissue fragments were transferred to a recording chamber and continuously perfused with Locke’s solution, maintaining a temperature of 37.0 ± 0.5°C. The temperature was monitored using a thermistor located adjacent to the recorded cell. For the single-cell suction pipette recording, the outer segment of the rod or cone was sucked into a glass pipette, containing a pipette solution consisting of 140 mM NaCl, 3.6 mM KCl, 2.4 mM MgCl2, 1.2 mM CaCl2, 0.02 mM EDTA, 10 mM glucose, and 3 mM HEPES, adjusted to pH 7.4. The pipette tip openings were approximately 2.5 μm in size, allowing for a tight fit with the outer segments. Light stimulation wavelengths of 500 nm, 520 nm, and 440 nm were respectively used for rod, M-cone, and S-cone recordings, Light stimulation durations were typically 10 ms and monochromatic in nature.

### 2.7 Image acquisition

All images were acquired using a Nikon A1R (Nikon Instruments Inc., Melville, New York) confocal microscope. ImageJ (RRID : SCR_003070) was used for creating composite figures of confocal images. IGOR Pro was used to render ERG figures (RRID : SCR_000325).

### 2.8 Statistical analysis

All data reported here were compiled and analyzed using MATLAB (Mathworks, RRID : SCR_001622).

## 3 Results

### 3.1 Rod pathway and AII amacrine cells in the ground squirrel retina

**Figure 1:**
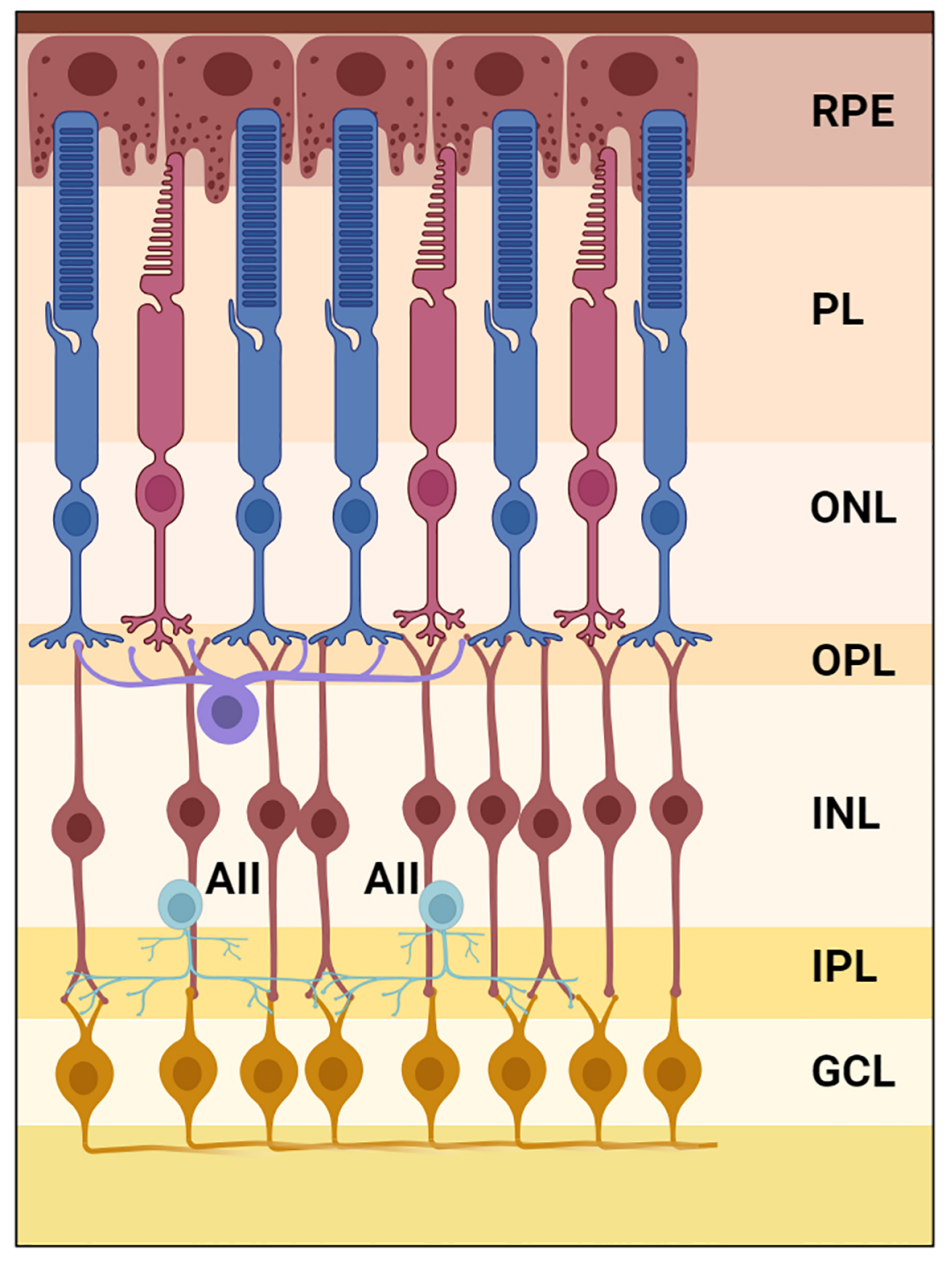
Illustration of the scotopic visual pathway in the TLGS retina created using BioRender.com. The image depicts the synaptic connectivity of AII-amacrine cells (light blue) at the outer boundaries of the inner plexiform layer. RPE, retinal pigment epithelium; PL, photoreceptor layer; ONL, outer nuclear layer; OPL, outer plexiform layer; INL, inner nuclear layer; AII, AII amacrine cells; IPL, inner plexiform layer; GCL, ganglion cell layer.

**Figure 2:**
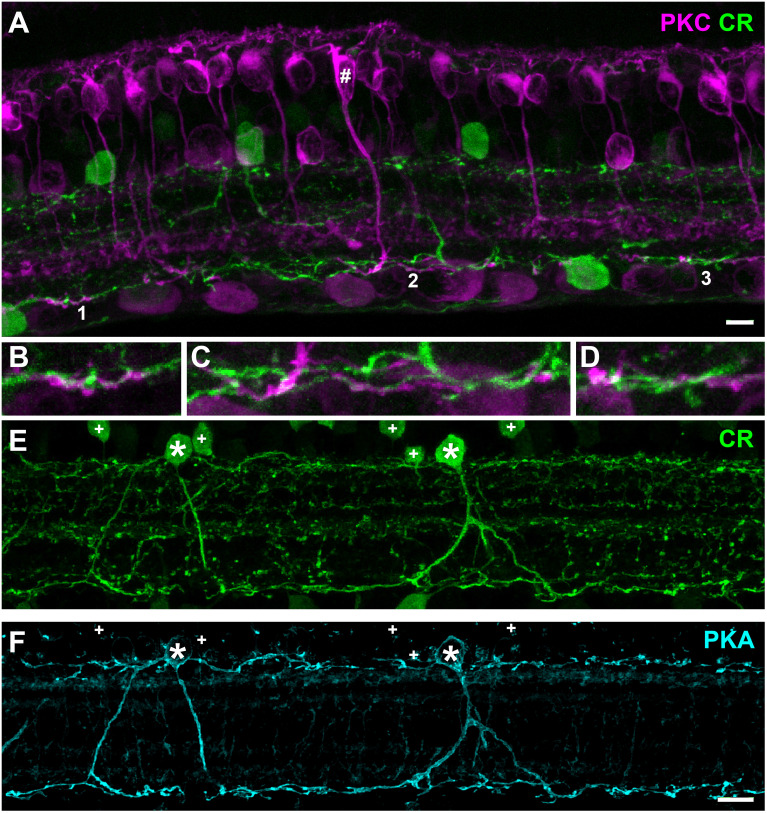
Retinal slice stained with protein kinase C (PKC) and calretinin (CR). **(A)** A RBC (#) is stained with PKC (magenta) sending an axon terminal to the bottom of IPL. Putative AIIs are labeled with an antibody against calretinin. **(B–D)** zoomed view of areas 1, 2, and 3 in **(A)** show intertwined RBC axonal terminals and AII dendrites at the bottom of IPL. **(E)** TLGS retinal cell layers stained with calretinin indicate sparse AII morphology. “+” denotes soma of other non AII amacrine cells labeled with calretinin. **(F)** Same image as shown in **(E)** above but using PKA antibody to specifically label AII amacrine cells. Note “+” denotes calretinin positive amacrine somas that were not labeled by PKA. In all images * indicates rod bipolar cells. Scale bars: 5 µm.

**Figure 3:**
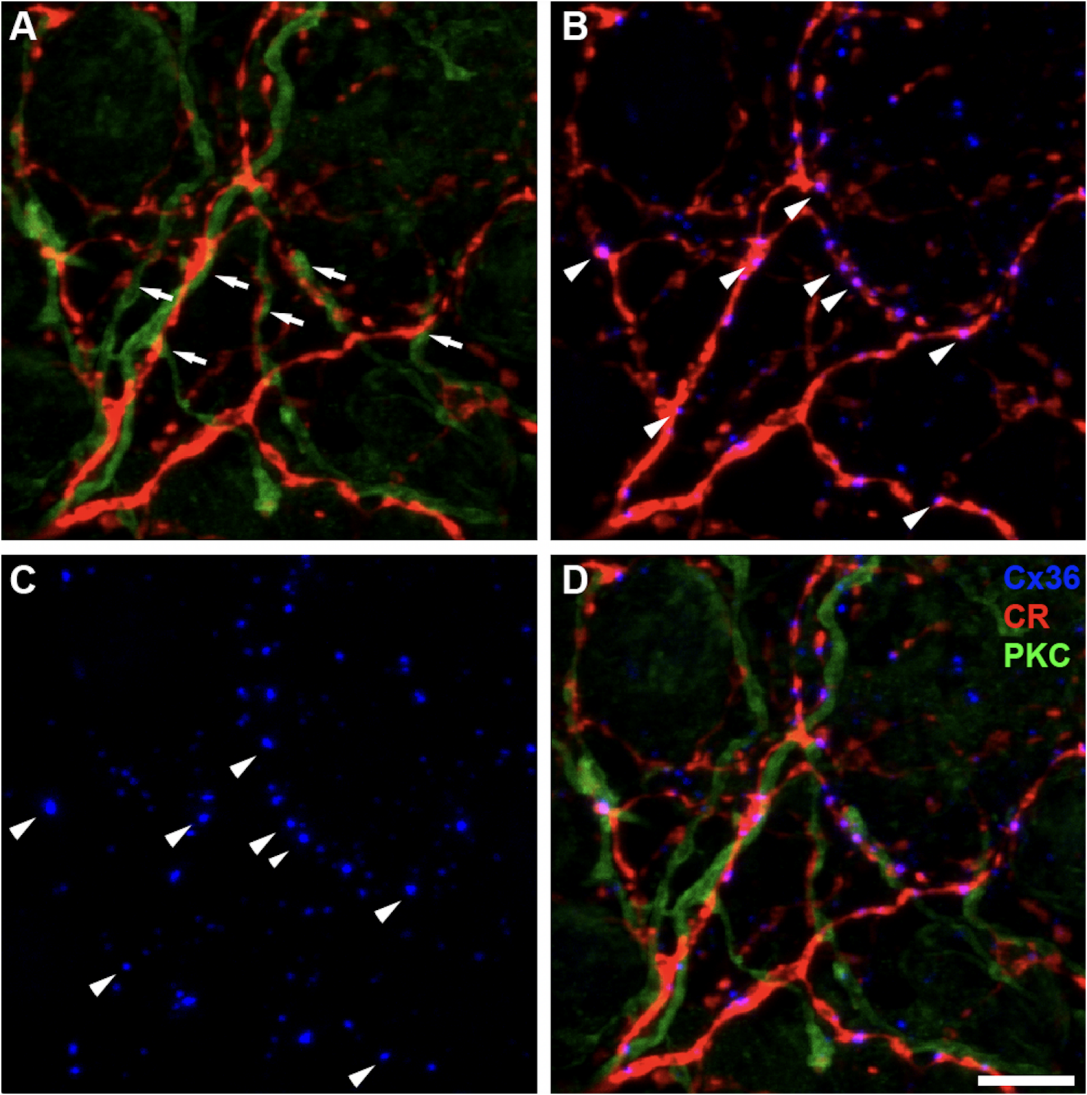
**(A)** Whole-mount retina showing AII-amacrine processes stained with calretinin (CR, red) and RBC processes stained with protein kinase C (PKC, green). Areas of putative AII-RBC synaptic activity are annotated (white arrows). **(B)** Calretinin^+^ AII-amacrine processes (red) co-labeled with Cx36 (blue) a retinal gap junction subunit. Colocalization of Cx36 puncta along AII amacrine processes indicate AII-AII synaptic activity or AII-ON bipolar cell gap junctions. Arrow heads indicate selected examples of sites of superposition. **(C)** Same image as shown in **(B)** with only Cx36 puncta labeling (blue). **(D)** Superimposed image of AII (CR, red), RBC (PKC, green) and Cx36 puncta (Cx36, blue).

Most mammals are rod-dominant, and their retinas contain a type of bipolar cells that specifically contact rods, with occasional cone connections [**Figure 1**]. With about 15% rod photoreceptors, we speculate that a type of rod-specific bipolar cells might be retained in the TLGS retina. Rod bipolar cells (RBC) can be identified in almost all the mammalian retinas examined by their axonal arbors ramifying at the bottom of the inner plexiform layer (IPL). Additionally, they can often be labeled by PKC-alpha antibodies. Individual rod bipolar cells in retinal slices of TLGS have been injected with neurobiotin, which were subsequently visualized with fluorescent dye and an antibody against PKC-alpha (8). A small population of bipolar cells have a very large dendritic expansion reaching out to sparse photoreceptors and an equally large size of axonal arbor ramifying at the border of IPL and ganglion cell layer (GCL). Such cells are always positive for PKC-alpha staining (8) and are thus likely RBCs, although some of the PKC-alpha positive cells are cone bipolar cells with axon terminal ramifying in the middle of IPL (**Figure 2A**, a RBC with axon terminal reaching the bottom of IPL was marked by #). We also labeled the retinal slices with an antibody against calretinin (**Figure 2**), a protein frequently detected in AIIs in other mammalian retinas. We observed that one type of calretinin-positive amacrine cells have an upper tier of dendrites along the boundary of inner nuclear layer (INL) and the IPL (**Figure 2E**, asterisk), while sending descending processes down to the bottom of IPL (**Figures 2B–D**, zoomed view of areas 1-3 in **Figure 2A**), a familiar pattern seen in other mammalian species. Note that in addition to putative AIIs, calretinin also faintly labels other amacrine cells (**Figures 2E, F**, +) with dendrites in the middle of IPL (**Figure 3E**). A PKA antibody seems to label only AII amacrine cells (**Figure 3F**), although it also labels many bipolar cells (not shown in this frame). The axon terminals of RBC and the dendrites of AIIs are in close opposition at the bottom of IPL, likely form synapses as observed in other species (3). Similar contacts can be better visualized in a wholemount view (**Figure 3A**). Note that the dendritic sprawl of such putative AII cells is markedly increased compared to the normal morphology of rod-dominant eyes. Thus, it seems that cellular components of the rod pathway are preserved in this cone-dominant retina. Importantly, both RBCs and AIIs appear to be much more sparse than in mouse, rabbit, and non-human primate retinas (**Figure 2E**), matching the low proportion of rod photoreceptors in TLGS.

### 3.2 Similar to rod-dominant species AIIs in TLGS are connected by gap junctions

One of the hallmark of AIIs in mammalian retinas is that they are extensively connected by gap junctions forming an extensive neural network, whose conductance can be modulated by the state of light adaptation (13–15). Connexin 36 (Cx36) is the molecular constituent of AII-AII gap junctions, and Cx36 channels on AIIs account for the majority of Cx36 proteins in the inner retina (16). Accordingly, we observed numerous Cx36 puncta on AII processes (**Figures 3B-D**), and most of the Cx36 puncta at this layer of IPL are in fact on AII processes, similar to what has been reported in mammalian retinas (16). This further supports the notion that these calretinin-positive amacrine cells with processes at the bottom of IPL are very likely AIIs.

### 3.3 Single cell recordings of photoreceptors and electroretinography

**Figure 4:**
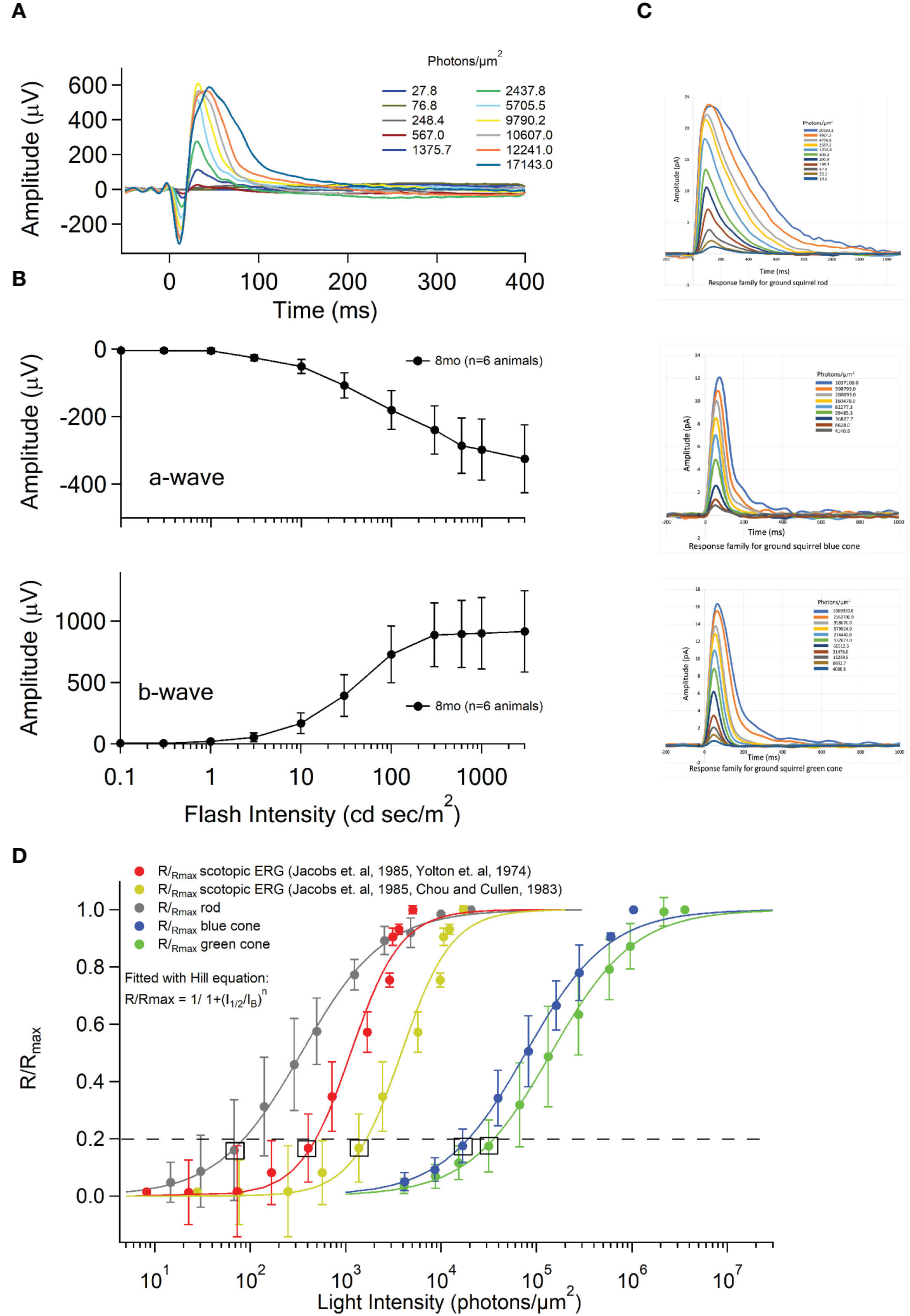
Dark-adapted *in vivo* ERG and single cell suction electrode recordings from rod and cone outer segments. **(A)** Scotopic ERG flash response family (n=6 TLGS) to binocular full-field ganzfeld stimulation. The flash intensity of the xenon (white) stimulus ranged from 0.1 to 3000 cd sec/m^2^. The time t=0ms indicates the time at the onset of the 4 ms xenon flash. Response traces are arbitrarily colored and corresponding flash strengths for each stimulus are shown to the right. Values have been converted from cd sec/m^2^ to photons/µm^2^ to facilitate comparison. **(B)** Average ERG a-wave and b-wave amplitudes as a function of flash intensity in 8mo old TLGS (n=6 squirrels). The a-wave amplitude is measured from the pre-stimulus baseline to the first negative trough. The b-wave amplitude is measured from the trough of the a-wave to the most positive peak following the a-wave. Error bars represent standard deviation. **(C)** Upper plot, dark-adapted flash response family from single-cell suction electrode recordings of TLGS rods (n=4, from 2 animals). Membrane current response of rod outer segments to a series of 10 ms flashes (500 nm) of increasing intensity from 15 to 20,000 photons/um^2^. Middle plot, dark-adapted flash response family from single cell suction electrode recordings of TLGS S-cones (n=4, from 3 animals). Membrane current responses of S-cone outer segments to a series of 10 ms flashes (440 nm) of increasing intensity from 4000 to 1M photons/um^2^. Bottom plot, dark-adapted flash response family from single cell suction electrode recordings of TLGS M-cones (n=8, from 6 animals). Membrane current response of green cone outer segments to a series of 10 ms flashes (520 nm) of increasing intensity from 4000 to 3.5M photons/um^2^. **(D)** Intensity-Response curves for single cell suction electrode recordings of TLGS M-cones (green), S-cones (blue), and rods (gray) plotted in comparison to the *in vivo* scotopic ERG intensity-response (red and yellow). Data were fit with a Hill equation: R/Rmax = 1/1+(I_1/2_/I_B_)^n^. To facilitate comparison, the boxes indicate flash intensity that elicits a response just below a detection threshold arbitrarily set at 20% of Rmax. Satisfying this requirement, for rod photoreceptors the closest R/Rmax=0.16 +/- 0.13 (n=3 rod photoreceptors, n=2 animals) elicited by a flash (I_F_) of 67.8 photons/µm^2^. Hill coefficient n=0.98, Half-saturating light intensity I_1/2 =_ 342.9 photons/µm^2^. For S-cone photoreceptors the closest R/Rmax=0.18 +/-0.06 (n=4 blue cones, n=3 animals) at an I_F_ of 16827.7 photons/µm^2^. Hill coefficient n=1.05, Half-saturating light intensity I_1/2 =_ 77488.2 photons/µm^2^. For M-cone photoreceptors the closest R/Rmax=0.17 +/-0.09 (n=9 green cones, n=6 animals) at an I_F_ of 31476.01 photons/µm^2^. Hill coefficient n=1.00, Half-saturating light intensity I_1/2 =_ 142536 photons/µm^2^. For *in vivo* scotopic ERGs the light intensities estimated at the retina varied by ~250 photons/um^2^ depending on assumptions regarding preretinal optical absorption (18–20). Using the GS spectral sensitivity curve (11) and the preretinal absorbance in GS described in Yolton et. al, 1974 the closest R/Rmax=0.17+/-0.07 (n=6 animals) at an I_F_ of 1375.7 photons/µm^2^ (12). Hill coefficient n=1.65, Half-saturating light intensity I_1/2 =_ 3831.4 photons/µm^2^. Using the GS spectral sensitivity curve (11) and the spectral transmittance measurements of the ocular media of the GS described in Chou and Cullen, 1983 the closest R/Rmax=0.17+/-0.07 (n=6 animals) at an I_F_ of 405.4 photons/µm^2^. Hill coefficient n=1.65, Half-saturating light intensity I_1/2 =_ 1128.7 photons/µm^2^ (20, 21).

The electroretinogram (ERG) of the ground squirrel has been reported with controversial results regarding the contribution of rods to the scotopic ERG (9, 17). We recorded scotopic ERG from dark-adapted TLGS and compare it with single cell light responses of rods, M-cones, and S-cones using suction electrode method. We found that the threshold for a single TLGS rod is about 10 photons per μm^2^ per 10ms, where the threshold for a single TLGS cone is about 10000 photons per μm^2^ per 10ms (**Figure 4D**). In comparison, the threshold for scotopic ERG was calculated to be at least 10 times more sensitive than the cone threshold (**Figure 4**). Thus, it is likely that both rods and cones contribute to the scotopic ERG and the rod pathway in the TLGS is functional.

## 4 Discussion

AII-amacrine cells and their role in scotopic visual pathways in TLGS have remained largely uncharacterized despite their conserved role across mammalian species. In confocal imaging of the AII-amacrine cell in the cone-dominant retina, we noticed a marked shift in AII cell morphology and density compared to their rod-dominant counterparts. AIIs in TLGS retina have long processes in both upper and lower border of IPL, connected by vertical thick stalk from the soma. They are sparsely distributed, unlike in rod-dominant retinas. Like AIIs in rod-dominant retinas, Connexin36 are densely expressed on AII cell dendrites. Similarly, while Connexin36 is primarily expressed by the AII cell, it is also sparsely expressed in processes from other cell types across the IPL, as well as in the outer retinal layers by photoreceptors (13, 16). This low density of AIIs and widely expanded dendritic field match the small population of rods in TLGS retina, suggesting AIIs in TLGS may serve chiefly the rod pathway, but do not contribute significantly to cone pathways, especially ones encoding higher spatial resolution information. A recent study on the evolutionary origin of the rod bipolar cell pathway in the vertebrate retina reports AII-like amacrine cells associated with ancient rod bipolar cells in the fish retina (17), suggesting AIIs are likely co-evolved with RBCs in early vertebrates before mammals.

Another unsettled question regarding rod vision in this cone-dominant retina has been the contribution of rods to scotopic ERG (9, 17, 22). By using light stimuli of different wavelengths, it has been reported that the contribution of rods to scotopic ERG was variable. By directly comparing rod and cone single cell sensitivity with scotopic ERG recordings, here we found that TLGS rods are comparable in their sensitivity with rods from other mammalian species. However, the small population of rods render their contribution to the scotopic ERG responses reduced. Another question is why TLGS and other cone-dominant mammals, such as tree shrews, have evolved to maintain a small population of rods (23). Studies on ambient light entrainment suggest AII cells and the conserved rod pathway may share connectivity with ipRGCs (18, 24) and provide photic input to the body’s master clock, the suprachiasmatic nucleus (SCN). Potential additional roles for the conserved rod pathway may contribute to ambient light entrainment and connectivity with ipRGCs (18, 24). Other mammals, like the *Tupaia glis* or tree shrew, also have been proposed to similarly conserve a functional rod pathway despite being a cone-dominant eye, in which AII- amacrine cells, and the rod pathway more broadly, could serve non-image forming functions (19), a proposition to be examined.

## 5 Conclusion

The TLGS rod pathway is largely conserved in comparison to mouse, rabbit, and non-human eyes despite being a cone-dominant retina. The role of AII amacrine cells specifically are correlated with a decrease in density to accompany the relatively smaller population of rods and rod bipolar cells in the cone-dominant TLGS eye. Also, AII amacrine cells appear to be connected by gap junctions, suggesting an evolutionary conserved feature of the rod pathway. TLGS retain scotopic vision, however, their weakened contribution to scotopic vision may indicate additional roles for the conserved pathway in the cone-dominant mammalian eye.

## Data availability statement

The raw data supporting the conclusions of this article will be made available by the authors, without undue reservation.

## Ethics statement

TLGS were housed and maintained according to their protocol ASP#595, approved by the National Institute of Health guidelines for Animal Care and Use Committee in research and by the Ethical and Animal Studies Committee of the National Eye Institute.

## Author contributions

RF: Data curation, Formal Analysis, Investigation, Writing – original draft, Writing – review & editing. KM: Conceptualization, Data curation, Formal Analysis, Investigation, Methodology, Project administration, Resources, Software, Supervision, Validation, Visualization, Writing – original draft, Writing – review & editing. FN-N: Investigation, Methodology, Project administration, Writing – review & editing. WL: Conceptualization, Data curation, Formal Analysis, Funding acquisition, Investigation, Methodology, Project administration, Resources, Software, Supervision, Validation, Visualization, Writing – original draft, Writing – review & editing.
